# An exploration into the effect of foot orthoses on bone marrow lesions associated with mechanical foot pain

**DOI:** 10.1186/1757-1146-7-S2-A1

**Published:** 2014-11-14

**Authors:** Jill Halstead, Anne-Maree Keenan, Dennis McGonagle, Philip Conaghan, Anthony Redmond

**Affiliations:** 1 University of Leeds, NIHR Leeds Musculoskeletal Biomedical Research Unit, Leeds, UK

## Background

Mechanical foot pain is a syndrome in which local pain is associated with musculoskeletal tissues, occurring as a result of mechanical stress. Recent research has indicated that bone marrow lesions (BML) in the knee are associated with mechanical knee pain and increased forces, and that changing the forces within the knee can alter the pattern of BML. As foot orthoses are thought to act by modifying abnormal foot motion and internal distribution of forces, the aim of this study was to explore whether foot orthoses can modify volumes and patterns of BML in patients with mechanical midfoot pain.

## Methods

Forty-two patients were recruited from the Leeds Musculoskeletal Podiatry Service with mechanical midfoot pain and presence of BML as identified on 0.2T extremity magnetic resonance (MR) imaging. Patients were randomised to receive either (i) and active intervention, a pre-fabricated foot orthoses (VectOrthotic® Healthy Step, Sensograph Ltd) or (ii) a sham device, which consisted of 4mm of flat, compressed, closed-cell polyethelene foam. A group of age matched controls (people with no mechanical foot pain) was also recruited (n=41). Outcome data included patient reported outcomes and high resolution MR images, captured using a 3T scanner. Patients were seen at 6 weeks, and then three months after receiving the orthoses, when measures were repeated.

## Results

As this was an exploratory study, no formal inferential analysis was undertaken. At baseline, there were some incidental BML identified in the control group (n=3 lesions), the mechanical foot pain group demonstrated 108 BMLs, with the most common sites being the cuneiforms and the second metatarsal. After 6 weeks, there was a greater reduction in the mean visual analogue pain scale in the active intervention (-14.81mm, CI -22.3 to -7.3) compared to the sham (-7.37mm, CI -19.9 to 5.2) intervention group, and in the active group (pre-formed devices) there was a further reduction in pain (-7.07mm, CI -15 to 0.9) from 6 to 12 weeks that was not shown in the sham group (+2.81mm, CI -9.1 to 14.7). By 3 months, the mean BML volume was decreased by 26% in the pre-fabricated orthoses group, compared to a reduction of only 4% in the sham group. Furthermore, BML patterns were altered systematically in the pre-fabricated orthoses group with a greater reduction in medial bones with BML, while the sham group remained relatively unchanged.

## Conclusion

Our data are consistent with other published research reporting that patient reported outcomes are improved in patients wearing foot orthoses. We also found a higher frequency of BML in people with mechanical foot pain. More importantly, the MRI data indicate that with the use of prefabricated orthoses we were able to reduce BML volume (a measure of bone response to mechanical stress) and to alter systematically the pattern of BML in the tarsal bones. These results suggest that functional foot orthoses do have an effect on the bone stress continuum and provide further support to the hypothesis that functional orthoses act through internal distribution of forces within the foot.

